# Reduced Risk of Reinfection with SARS-CoV-2 After COVID-19 Vaccination — Kentucky, May–June 2021

**DOI:** 10.15585/mmwr.mm7032e1

**Published:** 2021-08-13

**Authors:** Alyson M. Cavanaugh, Kevin B. Spicer, Douglas Thoroughman, Connor Glick, Kathleen Winter

**Affiliations:** ^1^Epidemic Intelligence Service, CDC; ^2^Kentucky Department for Public Health; ^3^Division of Healthcare Quality Promotion, National Center for Emerging and Zoonotic Infectious Diseases, CDC; ^4^Division of State and Local Readiness, Center for Preparedness and Response, CDC; ^5^College of Public Health, University of Kentucky, Lexington, Kentucky.

Although laboratory evidence suggests that antibody responses following COVID-19 vaccination provide better neutralization of some circulating variants than does natural infection (*1*,*2*), few real-world epidemiologic studies exist to support the benefit of vaccination for previously infected persons. This report details the findings of a case-control evaluation of the association between vaccination and SARS-CoV-2 reinfection in Kentucky during May–June 2021 among persons previously infected with SARS-CoV-2 in 2020. Kentucky residents who were not vaccinated had 2.34 times the odds of reinfection compared with those who were fully vaccinated (odds ratio [OR] = 2.34; 95% confidence interval [CI] = 1.58–3.47). These findings suggest that among persons with previous SARS-CoV-2 infection, full vaccination provides additional protection against reinfection. To reduce their risk of infection, all eligible persons should be offered vaccination, even if they have been previously infected with SARS-CoV-2.*

Kentucky residents aged ≥18 years with SARS-CoV-2 infection confirmed by positive nucleic acid amplification test (NAAT) or antigen test results^†^ reported in Kentucky’s National Electronic Disease Surveillance System (NEDSS) during March–December 2020 were eligible for inclusion. NEDSS data for all Kentucky COVID-19 cases were imported into a REDCap database that contains laboratory test results and case investigation data, including dates of death for deceased patients reported to public health authorities (*3*). The REDCap database was queried to identify previously infected persons, excluding COVID-19 cases resulting in death before May 1, 2021. A case-patient was defined as a Kentucky resident with laboratory-confirmed SARS-CoV-2 infection in 2020 and a subsequent positive NAAT or antigen test result during May 1–June 30, 2021. May and June were selected because of vaccine supply and eligibility requirement considerations; this period was more likely to reflect resident choice to be vaccinated, rather than eligibility to receive vaccine.^§^ Control participants were Kentucky residents with laboratory-confirmed SARS-CoV-2 infection in 2020 who were not reinfected through June 30, 2021. Case-patients and controls were matched on a 1:2 ratio based on sex, age (within 3 years), and date of initial positive SARS-CoV-2 test (within 1 week). Date of initial positive test result refers to the specimen collection date, if available. The report date in NEDSS was used if specimen collection date was missing. Random matching was performed to select controls when multiple possible controls were available to match per case (*4*).

Vaccination status was determined using data from the Kentucky Immunization Registry (KYIR). Case-patients and controls were matched to the KYIR database using first name, last name, and date of birth. Case-patients were considered fully vaccinated if a single dose of Janssen (Johnson & Johnson) or a second dose of an mRNA vaccine (Pfizer-BioNTech or Moderna) was received ≥14 days before the reinfection date. For controls, the same definition was applied, using the reinfection date of the matched case-patient. Partial vaccination was defined as receipt of ≥1 dose of vaccine, but either the vaccination series was not completed or the final dose was received <14 days before the case-patient’s reinfection date. Using conditional logistic regression, ORs and CIs were used to compare no vaccination and partial vaccination with full vaccination among case-patients and controls. SAS (version 9.4; SAS Institute) was used for matching and statistical analyses. This activity was reviewed by CDC and was conducted consistent with applicable federal law and CDC policy.^¶^

Overall, 246 case-patients met eligibility requirements and were successfully matched by age, sex, and date of initial infection with 492 controls. Among the population included in the analysis, 60.6% were female, and 204 (82.9%) case-patients were initially infected during October–December 2020 (Table 1). Among case-patients, 20.3% were fully vaccinated, compared with 34.3% of controls (Table 2). Kentucky residents with previous infections who were unvaccinated had 2.34 times the odds of reinfection (OR = 2.34; 95% CI = 1.58–3.47) compared with those who were fully vaccinated; partial vaccination was not significantly associated with reinfection (OR = 1.56; 95% CI = 0.81–3.01).

**TABLE 1:** Demographic characteristics of COVID-19 patients with reinfection (case-patients) and COVID-19 patients who were not reinfected (control participants) — Kentucky, May–June 2021

Characteristic	No. (%)	
	Case-patients* (n = 246)	Control participants^†^ (n = 492)
**Age group, yrs**		
18–29	46 (18.7)	89 (18.1)
30–39	37 (15.0)	83 (16.9)
40–49	43 (17.5)	80 (16.3)
50–59	44 (17.9)	88 (17.9)
60–69	27 (11.0)	51 (10.4)
70–79	28 (11.4)	58 (11.8)
≥80	21 (8.5)	43 (8.7)
**Sex**		
Female	149 (60.6)	298 (60.6)
**Month of initial infection in 2020**		
March	0 (0)	3 (0.6)
April	7 (2.8)	11 (2.2)
May	2 (0.8)	2 (0.4)
June	4 (1.6)	11 (2.2)
July	8 (3.3)	17 (3.5)
August	8 (3.3)	13 (2.6)
September	13 (5.3)	22 (4.5)
October	36 (14.6)	78 (15.9)
November	72 (29.3)	141 (28.7)
December	96 (39.0)	194 (39.4)

* Case-patients were eligible for inclusion if initial infection occurred during March–December 2020, and a subsequent positive nucleic acid amplification or antigen test result was received during May–June 2021 (using date of specimen collection). Cases for analyses were restricted to persons aged ≥18 years at time of reinfection.

**^†^** Controls were matched by sex, age (within 3 years), and time of initial infection diagnosis (within 7 days).

**TABLE 2:** Association of SARS-CoV-2 reinfection* with COVID-19 vaccination status — Kentucky, May–June 2021

Vaccination status	No. (%)		OR (95% CI)^†^
	Case-patients	Control participants	
Not vaccinated	179 (72.8)	284 (57.7)	2.34 (1.58–3.47)
Partially vaccinated^¶^	17 (6.9)	39 (7.9)	1.56 (0.81–3.01)
Fully vaccinated^§^	50 (20.3)	169 (34.3)	Ref
**Total**	**246 (100)**	**492 (100)**	**—**

**Abbreviations:** CI = confidence interval; NAAT = nucleic acid amplification test; OR = odds ratio; Ref = referent group.

*All case-patients (reinfected) and control participants (not reinfected) had previous SARS-CoV-2 infection documented by positive NAAT or antigen test results during March–December 2020. Reinfection was defined as receipt of positive NAAT or antigen test results during May 1–June 30, 2021.

^†^ Estimated based on conditional logistic regression.

^§^ Case-patients were considered partially vaccinated if ≥1 dose of vaccine was received, but the vaccination series was either not completed or the final dose was received <14 days before their reinfection date. For control participants, the same criteria were applied, using the matched case-patient’s reinfection date.

^¶^ Case-patients and control participants were considered fully vaccinated if a complete COVID-19 vaccine series was received ≥14 days before the case-patient’s reinfection date.

## Discussion

This study found that among Kentucky residents who were previously infected with SARS-CoV-2 in 2020, those who were unvaccinated against COVID-19 had significantly higher likelihood of reinfection during May and June 2021. This finding supports the CDC recommendation that all eligible persons be offered COVID-19 vaccination, regardless of previous SARS-CoV-2 infection status.

Reinfection with SARS-CoV-2 has been documented, but the scientific understanding of natural infection-derived immunity is still emerging (*5*). The duration of immunity resulting from natural infection, although not well understood, is suspected to persist for ≥90 days in most persons.** The emergence of new variants might affect the duration of infection-acquired immunity, and laboratory studies have shown that sera from previously infected persons might offer weak or inconsistent responses against several variants of concern (*2*,*6*). For example, a recent laboratory study found that sera collected from previously infected persons before they were vaccinated provided a relatively weaker, and in some cases absent, neutralization response to the B.1.351 (Beta) variant when compared with the original Wuhan-Hu-1 strain (*1*). Sera from the same persons after vaccination showed a heightened neutralization response to the Beta variant, suggesting that vaccination enhances the immune response even to a variant to which the infected person had not been previously exposed. Although such laboratory evidence continues to suggest that vaccination provides improved neutralization of SARS-CoV-2 variants, limited evidence in real-world settings to date corroborates the findings that vaccination can provide improved protection for previously infected persons. The findings from this study suggest that among previously infected persons, full vaccination is associated with reduced likelihood of reinfection, and, conversely, being unvaccinated is associated with higher likelihood of being reinfected.

The lack of a significant association with partial versus full vaccination should be interpreted with caution given the small numbers of partially vaccinated persons included in the analysis (6.9% of case-patients and 7.9% of controls), which limited statistical power. The lower odds of reinfection among the partially vaccinated group compared with the unvaccinated group is suggestive of a protective effect and consistent with findings from previous studies indicating higher titers after the first mRNA vaccine dose in persons who were previously infected (*7*,*8*).

The findings in this report are subject to at least five limitations. First, reinfection was not confirmed through whole genome sequencing, which would be necessary to definitively prove that the reinfection was caused from a distinct virus relative to the first infection. Although in some cases the repeat positive test could be indicative of prolonged viral shedding or failure to clear the initial viral infection (*9*), given the time between initial and subsequent positive molecular tests among participants in this study, reinfection is the most likely explanation. Second, persons who have been vaccinated are possibly less likely to get tested. Therefore, the association of reinfection and lack of vaccination might be overestimated. Third, vaccine doses administered at federal or out-of-state sites are not typically entered in KYIR, so vaccination data are possibly missing for some persons in these analyses. In addition, inconsistencies in name and date of birth between KYIR and NEDSS might limit ability to match the two databases. Because case investigations include questions regarding vaccination, and KYIR might be updated during the case investigation process, vaccination data might be more likely to be missing for controls. Thus, the OR might be even more favorable for vaccination. Fourth, although case-patients and controls were matched based on age, sex, and date of initial infection, other unknown confounders might be present. Finally, this is a retrospective study design using data from a single state during a 2-month period; therefore, these findings cannot be used to infer causation. Additional prospective studies with larger populations are warranted to support these findings.

These findings suggest that among persons with previous SARS-CoV-2 infection, full vaccination provides additional protection against reinfection. Among previously infected Kentucky residents, those who were not vaccinated were more than twice as likely to be reinfected compared with those with full vaccination. All eligible persons should be offered vaccination, including those with previous SARS-CoV-2 infection, to reduce their risk for future infection.

**Summary**

**What is already known about this topic?**

Reinfection with human coronaviruses, including SARS-CoV-2, the virus that causes COVID-19, has been documented. Currently, limited evidence concerning the protection afforded by vaccination against reinfection with SARS-CoV-2 is available.

**What is added by this report?**

Among Kentucky residents infected with SARS-CoV-2 in 2020, vaccination status of those reinfected during May–June 2021 was compared with that of residents who were not reinfected. In this case-control study, being unvaccinated was associated with 2.34 times the odds of reinfection compared with being fully vaccinated.

**What are the implications for public health practice?**

To reduce their likelihood for future infection, all eligible persons should be offered COVID-19 vaccine, even those with previous SARS-CoV-2 infection.

## Footnotes

*
https://www.cdc.gov/vaccines/covid-19/clinical-considerations/covid-19-vaccines-us.html?CDC_AA_refVal=https%3A%2F%2Fwww.cdc.gov%2Fvaccines%2Fcovid-19%2Finfo-by-product%2Fclinical-considerations.html#CoV-19-vaccination

†
https://www.cdc.gov/coronavirus/2019-ncov/hcp/testing-overview.html

§ May and June were selected for two primary reasons. First, when vaccination supplies were low, some previously infected persons were deferring vaccination for 90 days to allow never-infected persons priority for available vaccine; however, by May 2021, deferral for 90 days was no longer a reason for those infected in 2020 to remain unvaccinated. Second, although vaccination eligibility was initially restricted based on age, comorbidities, and occupation, by April 5, 2021, all Kentucky residents aged ≥16 years became eligible for vaccination (https://chfs.ky.gov/agencies/dph/covid19/Cv19VaccineFAskedQ.pdf). Thus, vaccination status in May or June 2021 might more accurately reflect choice rather than eligibility to be vaccinated.

¶ 45 C.F.R. part 46.102(l)(2), 21 C.F.R. part 56; 42 U.S.C. Sect. 241(d); 5 U.S.C. Sect. 552a; 44 U.S.C. Sect. 3501 et seq.

**
https://www.cdc.gov/coronavirus/2019-ncov/hcp/duration-isolation.html
